# Combination of shear wave elastography and BI-RADS in identification of solid breast masses

**DOI:** 10.1186/s12880-021-00702-4

**Published:** 2021-12-01

**Authors:** Xue Zheng, Fei Li, Zhi-Dong Xuan, Yu Wang, Lei Zhang

**Affiliations:** 1 Pediatric Hospital Ultrasound Department, Cangzhou Central Hospital, Cangzhou, Hebei Province, China; 2 Department of Pulmonary and Critical Care Medicine (PCCM) Ward II, Cangzhou Central Hospital, Cangzhou, Hebei Province, China; 3 Department of Ultrasound III, Cangzhou Central Hospital, Cangzhou, Hebei Province, China; 4 Present Address: Department of Clinical Laboratory, Cangzhou Central Hospital, Cangzhou, Hebei Province, China

**Keywords:** BI-RADS, Shear wave elastography, Color doppler flow imaging, Solid breast mass, Diagnostic value

## Abstract

**Background:**

To explore the value of quantitative shear wave elastography (SWE) plus the Breast Imaging Reporting and Data System (BI-RADS) in the identification of solid breast masses.

**Methods:**

A total of 108 patients with 120 solid breast masses admitted to our hospital from January 2019 to January 2020 were enrolled in this study. The pathological examination served as the gold standard for definitive diagnosis. Both SWE and BI-RADS grading were performed.

**Results:**

Out of the 120 solid breast masses in 108 patients, 75 benign and 45 malignant masses were pathologically confirmed. The size, shape, margin, internal echo, microcalcification, lateral acoustic shadow, and posterior acoustic enhancement of benign and malignant masses were significantly different (all *P* < 0.05). The E mean, E max, SD, and E ratio of benign and malignant masses were significantly different (all *P* < 0.05). The E min was similar between benign and malignant masses (*P* > 0.05). The percentage of Adler grade II-III of the benign masses was lower than that of the malignant masses (*P* < 0.05). BI-RADS plus SWE yielded higher diagnostic specificity and positive predictive value than either BI-RADS or SWE; BI-RADS plus SWE yielded the highest diagnostic accuracy among the three methods (all *P* < 0.05).

**Conclusion:**

SWE plus routine ultrasonography BI-RADS has a higher value in differentiating benign from malignant breast masses than color doppler or SWE alone, which should be further promoted in clinical practice.

## Background

Mammography is the most widespread criterion for breast malignancy, while ultrasonography has a high value in screening high-density glandular tissue [1–3]. As the most potent method, B-mode ultrasound can show the morphological changes clearly with relatively ideal sensitivity, while it still has limitations due to a certain influence of subjective and objective factors. Color Doppler Flow Imaging (CDFI) can precisely show the condition of the blood vessels. The Breast Imaging Reporting and Data System (BI-RADS) based on CDFI has emerged as a powerful tool in the hemodynamic examination, thus playing a pivotal role in the diagnosis of solid breast masses [4, 5]. Shear wave elastography (SWE) is a new ultrasonic diagnostic technique, of which color-coded elastic hardness graph aids in the determination of tissue hardness, with high diagnostic value in differentiating benign and malignant prostate and thyroid [6, 7]. Based on this, we enrolled 108 patients with 120 solid breast masses admitted to our hospital from January 2019 to January 2020 to explore the diagnostic value of BI-RADS and SWE in solid breast masses, specifically as follows.

## Materials and methods

### General data

A total of 108 patients with 120 solid breast masses admitted to our hospital from January 2019 to January 2020 were enrolled in this study. The Ethics Committee of our hospital had approved the study protocol. The general data of the patients are shown in Table 1.

**Table 1:** General data of patients

	Cases	Age (Mean ± SD, years)	Types		
			Unilateral, Single	Bilateral, multiple	Unilateral, multiple
Female	104	52.56 ± 6.32	86	14	15
Male	4	53.21 ± 6.21	4	0	1

### Inclusion criteria

(1) Patients and their family members were fully informed of the study procedure and signed the Informed Consent Form; (2) Solid breast masses were confirmed by surgery or biopsy.

### Exclusion criteria

(1) Patients with mental problems or communication barriers; (2) Patients comorbid with other severe organic diseases.

### Ultrasonic examination

BI-RADS (Voluson P6, GE, American) was adopted, and the probe frequency was set to 12 MHz. The patient was places in supine position with the nipple as the center to perform radial examination. The detection depth was adjusted according to the lesions, and the position, size, shape, margin, internal echo, and other conditions of the solid breast masses were observed and recorded. Then the axillary lymph nodes were scanned. All patients underwent shear wave elastography (SWE) after locating the breast lesions. The lesions and surrounding normal glands and adipose tissue were included as possible in the elastic sampling frame, but skin and chest wall were excluded. Translucent dark blue represented the minimum hardness, red represented the maximum hardness; light blue, green, orange, and red increased with increasing hardness according to the approach of Versaci et al. [8]. The maximum and minimum values of elasticity were measured, and their mean values and ratios were calculated. All these indicators were measured 3 times and represented as E max, E min, E mean, and E ratio, respectively. The hemodynamics was detected by CDFI to comprehend the blood flow distribution inside the mass, and the Adler grading was adopted to record the degree of blood flow signals in tumors.

### Outcome measures

(1) **Pathological results.** Solid breast masses were defined as benign or malignant by pathological results. The benign masses included breast fibroadenoma, lobular adenomatous hyperplasia, papilloma, lipoma, and inflammatory masses, while the malignant masses included invasive ductal carcinoma invasive lobular carcinoma, and other masses. (2) **Two-dimensional gray-scale sonographic appearance.** The size, shape, margin, and internal echo of the benign and malignant masses were compared, and the number of microcalcification, lateral acoustic shadow, and posterior acoustic enhancement were collected. (3) **BI-RADS grading.** The masses were divided to six levels by BI-RADS. Level 0: Incomplete, which needs to be evaluated in conjunction with other methods; Level 1: Negative; Level 2: Benign; Level 3: Probably benign, which requires follow-ups; Level 4: Suspicious abnormality; Level 5: Highly suspicious of malignancy, which needs to be confirmed by puncture; Level 6: Known biopsy provrn malignancy. (4) **Ultrasonic elastography.** The diagnosis of a breast mass was determined by ultrasonic elastography based on the image properties of the area examined. Locally green stained areas around the lesion and uneven color inside the lesion (green and/or orange and red) were considered positive. (5) **Adler grading of blood flow.** Adler grading was used to classify blood flow detected into four grades: Grade 0: no blood flow; Grade I: a small amount of dot-like blood flow; Grade II: one longer blood flow through the mass, or 3 dot-like blood blood flow signals; Grade III: more than 4 dot-like blood blood flow signals [9–12].

### Statistics process

The enumeration data and measurement data were expressed as (Mean ± SD) and [n, (%)] and compared by t-test and chi-square test, respectively. SPSS20.0 was employed as the data processing software, and GraphPad Prism 7 was employed as the image rendering software. The difference was deemed statistically significant when P < 0.05.

## Results

### Pathological analysis of the masses

**Fig. 1:**
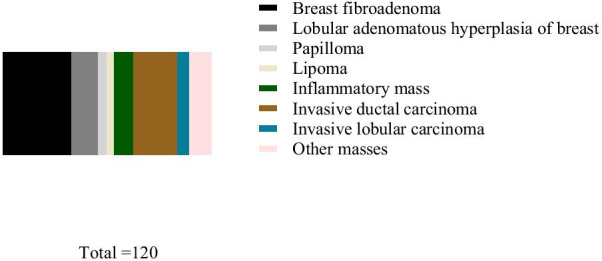
Pathological analysis of the masses. Benign masses: breast fibroadenoma (40 cases) in black, lobular adenomatoid hyperplasia (15 cases) in dark gray, papilloma (5 cases) in light gray, lipoma (4 cases) in yellow, and inflammatory mass (11 cases) in green. Malignant masses: invasive ductal carcinoma (25 cases) in brown, invasive lobular carcinoma (7 cases) in blue, and other masses (13 cases) in pink

Among the 120 solid breast masses, 75 benign and 45 malignant masses were pathologically confirmed. Among the benign masses, there were 40 cases of breast fibroadenoma, 15 cases lobular adenomatoid hyperplasia, 5 cases of papilloma, 4 cases of lipoma, and 11 cases of inflammatory mass. Among the malignant masses, there were 25 cases of invasive ductal carcinoma, 7 cases of invasive lobular carcinoma, and 13 cases of other masses (Fig. 1).

### Two-dimensional gray-scale sonographic appearance of benign and malignant breast masses

**Fig. 2:**
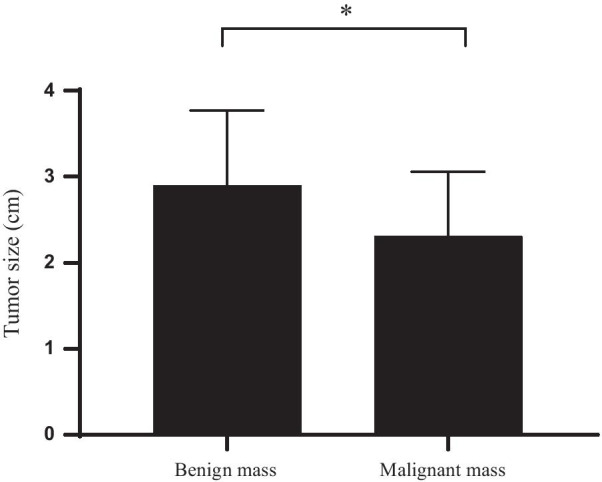
Comparison of the size of benign and malignant masses. The horizontal axis shows benign tumors and malignant tumors from left to right, and the vertical axis represents masses size (cm). The size of the benign masses was (2.90 ± 0.87) cm, and that of the malignant masses was (2.31 ± 0.75) cm

The size, shape, margin, internal echo, microcalcification, lateral acoustic shadow and posterior acoustic enhancement of benign and malignant masses were significantly different (all *P* < 0.05; Fig. 2 and Table 2–3).

**Table 2:** Two-dimensional gray scale sonographic appearance of benign and malignant breast masses

	Numbers	Shape		Margin		Internal Echo	
		Regular	Irregular	Sharp	Unsharp	Uniform	Nonuniform
Benign	75	50	25	54	21	58	17
Malignant	45	0*	45	2*	43	15*	30

^*^ Means *P* < 0.05 for comparison between groups

**Table 3:** Microcalcification, lateral acoustics and posterior acoustic enhancement of benign and malignant breast masses

	Numbers	Microcalcification	Lateral acoustics	Posterior acoustic enhancement
Benign	75	18	48	50
Malignant	45	39^*^	5^*^	5^*^

^*^ Means *P* < 0.05 for comparison between groups

### Comparison of BI-RADS and pathological results

BI-RADS confirmed 63 benign and 57 malignant masses in patients, with a diagnostic sensitivity of 93.33%, a diagnostic specificity of 80.00%, a positive predictive value of 73.68%, a negative predictive value of 95.23%, and a diagnostic accuracy of 85.00% (Table 4 and Table 8).

**Table 4:** Comparison of ultrasound and pathological results

		Pathological		
		Benign	Malignant	Total
BI-RADS	Benign	60	3	63
	Malignant	15	42	57
	Total	75	45	120

### Comparison of ultrasonic elastography parameters of benign and malignant breast masses

The E mean, E max, SD, and E ratio of benign and malignant masses were significantly different (all *P* < 0.05). The E min was similar in benign and malignant masses (Table 5).

**Table 5:** Comparison of ultrasonic elastography parameters of benign and malignant breast masses

	Benign (n = 75)	Malignant (n = 45)	*P*	t
E min	13.28 ± 5.58	14.05 ± 6.34	0.695	0.488
E mean	25.67 ± 8.23	62.25 ± 17.24	15.67	< 0.001
E max	48.68 ± 14.01	124.73 ± 30.79	18.47	< 0.001
SD	10.58 ± 3.28	30.25 ± 10.57	14.99	< 0.001
E ratio	2.36 ± 0.57	4.85 ± 2.05	9.923	< 0.001

### Comparison of SWE and pathological results

SWE confirmed 73 benign and 47 malignant masses, with a diagnostic sensitivity of 88.89%, a diagnostic specificity of 90.67%, a positive predictive value of 85.11%, a negative predictive value of 93.15%, and a diagnostic accuracy of 98.63% (Tables 6 and 8).

**Table 6:** Comparison of ultrasound and pathological results

		Pathological		
		Benign	Malignant	Total
SWE	Benign	68	5	73
	Malignant	7	40	47
	Total	75	45	120

### Comparison of BI-RADS plus SWE and pathological results

BI-RADS confirmed 73 benign and 47 malignant masses, with a diagnostic sensitivity of 97.78%, a diagnostic specificity of 96.00%, a positive predictive value of 93.62%, a negative predictive value of 98.63%, and a diagnostic accuracy of 96.67% (Tables 7 and 8).

**Table 7:** Comparison of ultrasound and pathological results

		Pathological		
		Benign	Malignant	Total
BI-RADS plus SWE	Benign	72	1	73
	Malignant	3	44	47
	Total	75	45	120

**Table 8:** Comparison of sensitivity, specificity, positive predictive value, negative predictive value, and diagnostic accuracy

	Sensitivity (%)	Specificity (%)	Positive predictive value (%)	Negative predictive value (%)	Diagnostic accuracy (%)
SWE	88.89	90.67	85.11	93.15	98.63
BI-RADS	93.33	80.00	73.68	95.23	85.00
BI-RADS plus SWE	97.78	96.00	93.62	98.63	96.67

### Comparison of diagnostic value of BI-RADS, SWE, and their combination

The diagnostic sensitivity, diagnostic specificity, positive predictive value, negative predictive value, and diagnostic accuracy of BI-RADS, SWE and BI-RADS plus SWE were shown in Table 8. BI-RADS plus SWE yielded higher diagnostic specificity and positive predictive value than BI-RADS and SWE alone; BI-RADS plus SWE yielded the highest diagnostic accuracy among the three methods (all *P* < 0.05).

### Comparison of Adler grading of benign masses in CDFI examination

**Fig. 3:**
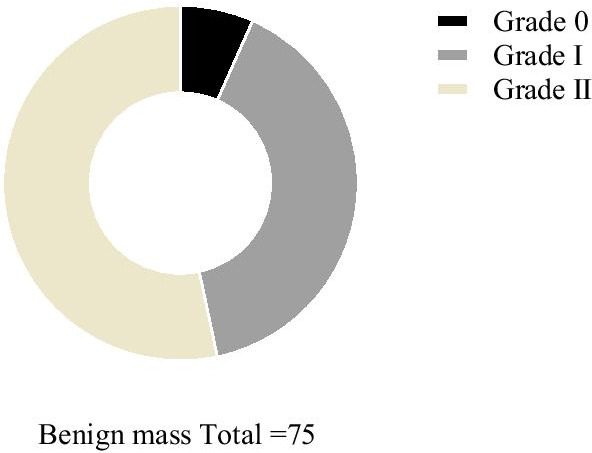
Comparison of Adler grading of benign masses in CDFI examination. Black area represents grade 0 (5), dark grey area represents grade I (30) and yellow area grade II (40). There were no grade III patients

**Fig. 4:**
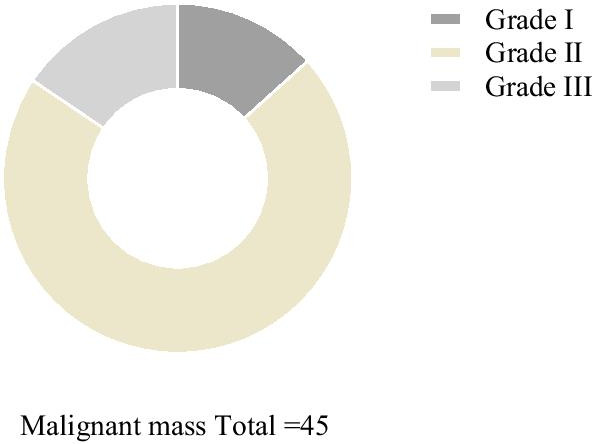
Comparison of Adler grading of malignant masses in CDFI examination. Dark grey area represents grade I (6), yellow area represents grade II (32), and light gray area represents grade III (7). There were no grade 0 patients

The percentage of Adler grade II-III of the benign masses (53.33%, 40/75) was lower than that of the malignant masses (86.67%, 39/45; *P* < 0.001; Figs. 3–4).

## Discussion

Mammography is the gold standard for breast cancer detection; whereas, some studies have shown that breast ultrasonography is increasingly important in the qualitative diagnosis of solid masses with a higher diagnostic value, in which two-dimensional ultrasonography plays a crucial role in the clinical diagnosis of malignant masses, which can clearly show the morphological characteristics [13–16]. In this study, the ultrasonogram of benign and malignant masses was significantly different regarding size, shape, margin, internal echo, microcalcification, lateral acoustic shadow and posterior acoustic enhancement. In addition, malignant masses are characterized by irregular mass shape and no enhancement of posterior echo, which is significantly different from benign masses with clear margins and uniform echo. Among the 120 solid breast masses detected in this study, 75 benign and 45 malignant masses were confirmed by pathological examination, while 69 benign and 51 malignant masses were confirmed by ultrasonography. It suggests that all malignant masses present more than one characteristic of malignant masses; moreover, some benign masses also show malignant signs such as microcalcification, which may be caused by secondary calcification in inflammatory lesions, so careful screening should be conducted.

Although two-dimensional ultrasonography has many advantages, it needs to be supplemented by other methods to confirm the nature of masses due to the fact that the malignant masses have unclear morphological characteristics in the early-stage [17–20]. The advancing of ultrasound technology allows for a wide application of Color Doppler in numerous diseases; this approach has a high sensitivity to blood flow and can reflect the the hemodynamics of the lesions, with a promising clinical application prospect. It is generally believed in the academic circles that there are differences in vascular morphology among masses of different natures: malignant masses are usually rich in blood vessels, but most of them are neovascularized and immature, while benign masses have mature and complete blood vessels, laying the foundation to distinguish benign and malignant masses with a good clinical application prospect [21–25]. In this study, the percentage of Adler grade II-III of the benign masses was lower than that of the malignant masses (53.33% vs. 86.67%), indicating that CDFI can be a scientific basis to determine the nature of the masses. Noteworthily, some benign masses present identically rich blood flow signals, which probably lies in the hyperplasia stage of them, so the combination of two-dimensional images is necessary for judgment.

As a new ultrasonic diagnostic technology, SWE employs the slow-moving shear wave generated by the fast acoustic radiation force at various points in the tissue to measure the small changes in the shear wave propagation of different tissue hardness through the real-time ultra-high speed imaging technology, which makes up for the deficiency of conventional ultrasonography in differentiating benign and malignant breast masses. In this study, the E max, E mean, and E ratio of malignant masses were significantly greater than those of benign masses, while no significant difference was found in E min. It indicates that the malignant masses have a hard elastic hardness, while the benign lesions have a soft elastic hardness, and that the difference between SWE plus BI-RADS and BI-RADS alone in differentiating benign and malignant breast masses is statistically significant. Malignant masses contain more collagen fibers with uneven distribution, showing green or disorderly colored images; benign messes, on the contrary, have less collagen fibers with uniform distribution [26]. Malignant masses are associated with collagen cross-link, which increases the stiffness of the extracellular matrix and the adhesion of the masses. SWE can quickly and intuitively show the hardness of the tissue, thus increasing the diagnostic specificity, positive predictive value and diagnostic accuracy when combined with BI-RADS.

## Conclusion

Collectively, Color Doppler is effective in differentiating benign from malignant breast masses. Whereas, SWE plus routine ultrasonography BI-RADS has a higher value in determining the nature of breast masses, which is worth of clinical promotion.

## Data Availability

The datasets used and/or analysed during the current study are available from the corresponding author on reasonable request.

## Abbreviations

SWE: Shear wave elastography
BI-RADS: Breast Imaging Reporting and Data System
CDFI: Color Doppler Flow Imaging
SWE: Shear wave elastography

## References

[1] Watanabe T, Kaoku S, Ban K (2019). B-mode ultrasound diagnostic flowchart for solid breast masses -JABTS BC-01 study. Ultrasound Med Biol.

[2] Park AY, Kwon M, Woo OH (2019). A Prospective Study on the Value of Ultrasound Microflow Assessment to Distinguish Malignant from Benign Solid Breast Masses: Association between Ultrasound Parameters and Histologic Micro vessel Densities. Korean J Radiol.

[3] Tran C, Vo Q (2019). Acoustic Radiation Force Impulse (ARFI) assessment of differential diagnosis of malignant and benign solid breast masses. Ultrasound Med Biol.

[4] Mehmet Sidiki Yelda, Goya C, Adin M E. Contribution of sonoelastography to diagnosis in distinguishing benign and malignant breast masses: role of sonoelastography in breast masses. J Ultrasound Med. 2020. 10.1002/jum.15236 32083349

[5] Sheela JEJ, Rosaline S, Mugila ST, et al. UWB sierpinski fractal antenna for breast tumour detection using SAR. IOP Conference Series: Materials Science and Engineering, 2021, 1055(1):012063

[6] Garganese G, Bove S, Fragomeni S (2021). Real-time ultrasound virtual navigation in 3D PET/CT volumes for superficial lymph node evaluation: an innovative fusion examination. Ultrasound Obst Gynecol.

[7] Kim ST, Lee JH, Lee H (2018). Visually interpretable deep network for diagnosis of breast masses on mammograms. Phys Med Biol.

[8] Versaci M, Calcagno S, Morabito FC. Fuzzy geometrical approach based on unit hyper-cubes for image contrast enhancement. 2015 IEEE International Conference on Signal and Image Processing Applications (ICSIPA). IEEE, 2016.

[9] Price ER, Sickles EA, Silaja Y (2018). Use of the probably benign (BI BI - RADS RADS category 3) assessment for masses on breast MRI MRI : Is it transferable to general clinical practice?. Breast J.

[10] Pasynkov DV, Egoshin IA, Kolchev AA (2020). Grayscale color mapping with the mathematical analysis of an ultrasound image in the differential diagnosis of cystic and solid breast masses. Vestn Rentgenol Radiol.

[11] Wiacek A, Falomo E, Myers K, et al. Clinical feasibility of coherence-based beamforming to distinguish solid from fluid hypoechoic breast masses. 2018 IEEE International Ultrasonics Symposium (IUS). IEEE, 2018.

[12] Nasief HG, Ivan M (2019). A quantitative ultrasound-based multi-parameter classifier for breast masses. Ultrasound Med Biol.

[13] Fatima K, Masroor I, Khanani S. Probably benign solid breast lesions on ultrasound: need for biopsy reassessed. Asian Pac J Cancer Prevent: APJCP, 2018, 19(12). 10.31557/APJCP.2018.19.12.3467 PMC6428540 30583671

[14] Omar L, Gleason MK, Pfeifer CM (2018). Management of palpable pediatric breast masses with ultrasound characteristics of fibroadenoma: a more conservative approach. Am J Roentgenol.

[15] M, A A , D, E E , K, Q K , et al. Comparative sonographic review of benign and malignant breast masses. J Solid Tumors, 2018, 8(1).

[16] A M E S, A B E A, A E M A, et al. Imaging of intracystic papillary carcinoma. Curr Probl Diagnostic Radiol. 2019, 48(4):348–352. 10.1067/j.cpradiol.2018.05.001 30072190

[17] Ji Hyun Y, et al. Grayscale ultrasound radiomic features and shear-wave elastography radiomic features in benign and malignant breast masses. Ultraschall in der Medizin (Stuttgart, Germany: 1980), 2019. 10.1055/a-0917-6825 31703239

[18] Fujioka T, Katsuta L, Kubota K (2020). Classification of breast masses on ultrasound shear wave elastography using convolutional neural networks. Ultrason Imaging.

[19] Nasief HG, Rosado-Mendez IM, Zagzebski JA (2019). Erratum to 'A Quantitative Ultrasound-Based Multi-Parameter Classifier for Breast Masses' [Ultrasound Med Biol 45 (2019) 1603–1616] - ScienceDirect. Ultrasound Med Biol.

[20] Perretta T, Meucci R, Pistolese CA (2021). Ultrasound-guided laser ablation after excisional vacuum-assisted breast biopsy for small malignant breast lesions: preliminary results. Technol Cancer Res Treat.

[21] Shin SY, Soochahn, et al. Joint weakly and semi-supervised deep learning for localization and classification of masses in breast ultrasound images. IEEE Trans Med Imaging, 2018. 10.1109/TMI.2018.2872031 30273145

[22] Goncharova AB, Busko EA. Software implementation for diagnostic decision-making based on multiparametric ultrasonography parameters of breast masses. 2020.

[23] Kikuchi M , Hayashida T , Watanuki R , et al. Abstract P1-02-09: diagnostic system of breast ultrasound images using Convolutional Neural Network. Abstracts: 2019 San Antonio Breast Cancer Symposium December 10–14, 2019 San Antonio, Texas. 2020.

[24] Negi A, Raj ANJ, Nersisson R (2020). RDA-UNET-WGAN: an accurate breast ultrasound lesion segmentation using Wasserstein generative adversarial networks. Arab J Sci Eng.

[25] Payne A, Merrill R, Minalga E (2020). A breast-specific MR guided focused ultrasound platform and treatment protocol: first-in-human technical evaluation. IEEE Trans Biomed Eng.

[26] Hari S. Ultrasonography in the diagnosis of solid breast masses. Diagnos Intervent Imaging, 2018.

